# Supplementary material for: Empirical methods for controlling false positives and estimating confidence in ChIP-Seq peaks
Source: BMC Bioinformatics. 2008 Dec 5;9:523. doi: 10.1186/1471-2105-9-523 (PMC2628906; doi:10.1186/1471-2105-9-523)
Supplement: Additional File 1 — A variety of html documents from the USeq web site detailing the available applications, their best usage, output file type descriptions, command line menus, etc. [file 1471-2105-9-523-S1.zip › USeqUserGuides/JavaDoc.zip!/JavaDoc/util/bio/wrappers/BL2Seq.html]

BL2Seq


|  |  |  |
| --- | --- | --- |
| **Overview** \| **Package** \| **Class** \| **Use** \| **Tree** \| **Deprecated** \| **Index** \| **Help** | |  |
| PREV CLASS   **NEXT CLASS** | **FRAMES**    **NO FRAMES**     **All Classes** |  |
| SUMMARY: NESTED \| FIELD \| CONSTR \| METHOD | DETAIL: FIELD \| CONSTR \| METHOD |  |


---


## util.bio.wrappers Class BL2Seq

```
java.lang.Object
  util.bio.wrappers.BL2Seq
```

---

``` public class BL2Seq extends java.lang.Object ```

This is a bit of a hack from the SimpleBlast, expand as needed.

---

**Constructor Summary**

`BL2Seq(java.lang.String fullPathToBL2seq)`


| **Method Summary** | |
| --- | --- |
| `BL2SeqHit[]` | `blastFASTAFiles(java.io.File seq1File, java.io.File seq2File)`             Uses BLAST bl2seq program to align two sequences found in the seq files, won't work on windows? |
| `java.lang.String[]` | `getCommandArray()` |
| `double` | `getExpectation()` |
| `java.lang.String` | `getFullPathToBL2seq()` |
| `BL2SeqHit[]` | `getHits()` |
| `double` | `getRawScoreCutOff()` |
| `java.lang.String[]` | `getRawUnparsedResults()` |
| `int` | `getW()` |
| `BL2SeqHit[]` | `parseBl2SeqResults(java.lang.String[] data)`             This takes an array of BLAST bl2seq results and parses the info into an array |
| `static java.util.ArrayList` | `parseLine(java.lang.String line, java.util.regex.Pattern pat)` |
| `void` | `setExpectation(double e)` |
| `void` | `setFullPathToBL2seq(java.lang.String fullPathToBL2seq)` |
| `void` | `setGapExtensionCost(int e)` |
| `void` | `setRawScoreCutOff(double rawScoreCutOff)` |
| `void` | `setRawUnparsedResults(java.lang.String[] rawUnparsedResults)` |
| `void` | `setW(int w)` |

**Methods inherited from class java.lang.Object**

`equals, getClass, hashCode, notify, notifyAll, toString, wait, wait, wait`

**Constructor Detail**

### BL2Seq

```
public BL2Seq(java.lang.String fullPathToBL2seq)
```


**Method Detail**

### blastFASTAFiles

```
public BL2SeqHit[] blastFASTAFiles(java.io.File seq1File,
                                   java.io.File seq2File)
```

:   Uses BLAST bl2seq program to align two sequences found in the seq files, won't work on windows?

---


### parseBl2SeqResults

```
public BL2SeqHit[] parseBl2SeqResults(java.lang.String[] data)
```

:   This takes an array of BLAST bl2seq results and parses the info into an array

---


### parseLine

```
public static java.util.ArrayList parseLine(java.lang.String line,
                                            java.util.regex.Pattern pat)
```

---


### getExpectation

```
public double getExpectation()
```

---


### setExpectation

```
public void setExpectation(double e)
```

---


### getFullPathToBL2seq

```
public java.lang.String getFullPathToBL2seq()
```

---


### setFullPathToBL2seq

```
public void setFullPathToBL2seq(java.lang.String fullPathToBL2seq)
```

---


### getRawScoreCutOff

```
public double getRawScoreCutOff()
```

---


### setRawScoreCutOff

```
public void setRawScoreCutOff(double rawScoreCutOff)
```

---


### getRawUnparsedResults

```
public java.lang.String[] getRawUnparsedResults()
```

---


### setRawUnparsedResults

```
public void setRawUnparsedResults(java.lang.String[] rawUnparsedResults)
```

---


### getW

```
public int getW()
```

---


### setW

```
public void setW(int w)
```

---


### getCommandArray

```
public java.lang.String[] getCommandArray()
```

---


### getHits

```
public BL2SeqHit[] getHits()
```

---


### setGapExtensionCost

```
public void setGapExtensionCost(int e)
```


---


|  |  |  |
| --- | --- | --- |
| **Overview** \| **Package** \| **Class** \| **Use** \| **Tree** \| **Deprecated** \| **Index** \| **Help** | |  |
| PREV CLASS   **NEXT CLASS** | **FRAMES**    **NO FRAMES**     **All Classes** |  |
| SUMMARY: NESTED \| FIELD \| CONSTR \| METHOD | DETAIL: FIELD \| CONSTR \| METHOD |  |


---
